# 8例脾弥漫性红髓小B细胞淋巴瘤患者临床特征及预后分析

**DOI:** 10.3760/cma.j.issn.0253-2727.2022.12.009

**Published:** 2022-12

**Authors:** 兴利 张, 菁 罗, 佼佼 张, 丽 陈, 杨 沈, 红梅 易, 立权 范, 坚青 糜

**Affiliations:** 1 上海血液学研究所，国家医学基因重点实验室，国家转化医学研究中心，上海交通大学医学院附属瑞金医院血液科，上海 200025 Shanghai Institute of Hematology, State Key Laboratory of Medical Genomics, National Research Center for Translational Medicine at Shanghai, Ruijin Hospital Affiliated to Shanghai Jiao Tong University School of Medicine, Shanghai 200025, China; 2 上海交通大学医学院附属瑞金医院病理科，上海 200025 Department of Pathology, Ruijin Hospital Affiliated to Shanghai Jiao Tong University School of Medicine, Shanghai 200025, China; 3 上海交通大学医学院附属瑞金医院，上海血液病研究所骨髓细胞室，上海 200025 Shanghai Institute of Hematology, Ruijin Hospital Affiliated to Shanghai Jiao Tong University School of Medicine, Shanghai 200025, China

**Keywords:** 脾脏肿瘤, 淋巴瘤，B细胞, 诊断, 治疗, Spleen neoplasms, Lymphoma, B-cell, Diagnosis, Therapeutics

## Abstract

**目的:**

探究脾弥漫性红髓小B细胞淋巴瘤（SDRPL）患者的临床特征、疗效及预后。

**方法:**

回顾性分析2017年5月至2022年4月在上海交通大学医学院附属瑞金医院诊治的8例SDRPL患者的病历资料，对患者的临床特征、实验室检查结果、骨髓和脾脏病理、疗效与预后进行分析及总结。

**结果:**

8例患者中位年龄54（42～69）岁。确诊时均表现为明显的脾脏肿大，多有淋巴细胞升高，PET/CT显示脾脏代谢不高或轻度增高。临床分期均为Ⅳ期，累及脾脏、外周血和骨髓，未见外周淋巴结受累。肿瘤细胞胞质丰富，易见短毛刺状突起。脾脏病理可见形态均一的小淋巴细胞弥漫浸润脾窦及脾索，白髓萎缩。免疫表型缺乏特异性，多表达CD19、CD20、CD79α等B细胞抗原。中位随访35.5（4～60）个月，脾切除术联合或不联合免疫化疗可使7例患者获得长期生存，1例伴CCND3 P284A和MYC S146L突变的患者在脾切除术后1个月进展为B细胞幼淋巴细胞白血病（B-PLL），在随访16个月时死亡。

**结论:**

SDRPL患者以中老年为主，临床呈惰性病程。患者大多可获得长期生存，但进展为B-PLL的患者预后较差。

## Abstract (en)

**Objective:**

To investigate the clinical characteristics, response, and prognosis of splenic diffuse red pulp small B-cell lymphoma（SDRPL）.

**Methods:**

Eight cases of SDRPL were diagnosed and treated at Ruijin Hospital Affiliated to Shanghai Jiao Tong University School of Medicine, between May 2017 and April 2022. Data on the clinical features, laboratory results, bone marrow and spleen biopsy results, response, and prognosis were collected and analyzed.

**Results:**

The median age at diagnosis was 54 (42–69) years. Splenomegaly and lymphocytosis were present in all cases, and PET/CT revealed normal to slightly elevated splenic FDG uptake. All cases were in stage IV, with spleen, peripheral blood, and bone marrow but no proximal lymph nodes involved. The cytoplasm of neoplastic villous cells was abundant, and splenic pathology showed that small homogenous lymphocytes permeated the splenic sinus and splenic cord, and the white pulp atrophied. Immunohistochemistry was not typical, and B-cell markers including CD19, CD20 and CD79α were positive. After a median follow up of 35.5 (4–60) months, 7 cases were alive after splenectomy with or without chemoimmunotherapy. The patient with CCND3 P284A and MYC S146L mutation developed to B-cell prolymphocytic leukemia (B-PLL) 1 month after splenectomy and died at 16 months of follow-up.

**Conclusion:**

A rare indolent B-cell lymphoma that primarily affects the elderly, SDRPL. Most patients achieved long-term survival, but the prognosis of patients who progress to B-PLL was poor.

原发于脾脏的淋巴瘤非常少见，在淋巴瘤中的比例不足2％，通常是B细胞来源[1]。最常见的组织学类型包括脾边缘区淋巴瘤（SMZL）、毛细胞白血病（HCL）、脾弥漫大B细胞淋巴瘤、毛细胞白血病变异型（HCL-v）和脾弥漫性红髓小B细胞淋巴瘤（SDRPL），其中HCL-v和SDRPL在2016版WHO造血与淋巴组织肿瘤分类中被归为脾B细胞淋巴瘤/白血病，不能分类型[2]。

SDRPL是一种罕见的惰性非霍奇金淋巴瘤（NHL），在NHL中发病率<1％[3]，在已发表的文献中多以个案报道的形式讨论其临床及病理特征[4]–[6]。为进一步提高临床医师对该病的认识，我们收集了符合这一诊断的8例患者的临床及病理资料，现总结如下。

## 病例与方法

1. 病例：收集2017年5月至2022年4月在上海交通大学医学院附属瑞金医院诊治的8例SDRPL患者。收集患者的临床资料包括性别、年龄、临床表现、伴随疾病、临床分期、实验室检查、治疗方案和结局等。所有病例的诊断基于2016版WHO分类中所描述的SDRPL的诊断标准：患者外周血和骨髓中可见极性分布的绒毛样淋巴细胞，核仁多不可见；脾脏体积显著增大，可见形态均一的小淋巴细胞弥漫性浸润脾窦及脾索，白髓萎缩；肿瘤细胞的免疫表型为B细胞抗原CD19、CD20、CD79α阳性表达，CD5、CD10、CD25、CD123、Annexin-A1均阴性表达，CD103、CD11c阳性表达很少；排除其他恶性淋巴瘤如SMZL、HCL、HCL-v、慢性淋巴细胞白血病（CLL）、淋巴浆细胞淋巴瘤等。

2. 方法：取干透的外周血和骨髓涂片行瑞氏染色后用低倍镜和油镜计数和观察细胞形态。脾脏组织标本经固定、脱水、浸蜡、石蜡包埋、切片后行苏木素-伊红（HE）染色，封片；对脾脏组织切片行免疫组化标记；采用二代测序（NGS）方法进行靶向DNA测序。由上海血液学研究所测序平台及上海瑞昂生物有限公司的商业化靶向检测试剂盒测序组完成。

3. 疗效评价：目前暂无针对SDRPL的统一疗效评价标准。鉴于SDRPL和CLL在临床表现、累及部位等方面相似，故参考iwCLL的疗效评价标准[7]，分为完全缓解（CR）、部分缓解（PR）、疾病稳定（SD）、疾病进展（PD）。

4. 生存指标定义及随访：总生存（OS）期定义为确诊SDRPL至死亡或随访截止时间。采用查阅患者住院病历和电话随访的形式随访，随访截止时间为2022年4月30日，中位随访35.5（4～60）个月。

5. 统计学处理：定量资料用中位数（范围）表示。

## 结果

1. 临床特征及实验室检查：本组病例男7例，女1例，中位年龄54（42～69）岁。确诊时4例患者因体检发现白细胞升高就诊，3例因脾肿大致腹胀就诊，1例因乏力就诊；2例伴B症状；3例伴慢性乙型肝炎，其中1例乙型肝炎病毒（HBV）DNA拷贝数升高；1例有荨麻疹病史。所有患者临床分期均为Ⅳ期，累及脾脏、外周血和骨髓，均未见外周淋巴结受累；均表现为明显的脾脏肿大，2例累及脾门淋巴结（例2、4），1例累及胰腺周围淋巴结（例4），1例累及邻近肝组织（例3）；1例患者出现脾脏梗死（例7）；PET/CT显示脾脏代谢不高或轻度增高（例1、2、6、8）（表1）。

**表1:** 脾弥漫性红髓小B细胞淋巴瘤（SDRPL）患者的临床特征

例号	性别	年龄（岁）	分期	累及部位	治疗方案	疗效	总生存期（月）
1	男	42	Ⅳa	脾脏、外周血、骨髓	脾切除术、R-CVP	CR	4
2	男	50	Ⅳa	脾脏、外周血、骨髓、脾门淋巴结	脾切除术、R-CVP×8、R维持	CR	60
3	男	52	Ⅳa	脾脏、外周血、骨髓、肝脏	CVP、IR、R-ICE、脾切除术、R-CHOP、Len+Clb	SD	42
4^a^	男	52	Ⅳb	脾脏、外周血、骨髓、脾门淋巴结、胰腺周围淋巴结	脾切除术、R-CHOP、FC、Clb	PD	16
5	男	56	Ⅳa	脾脏、外周血、骨髓	脾切除术	PR	18
6	男	62	Ⅳb	脾脏、外周血、骨髓	脾切除术、R-CHOP×6	CR	59
7	男	69	Ⅳa	脾脏、外周血、骨髓	Clb、脾切除术、R-CVP×8、R维持	CR	47
8	女	69	Ⅳa	脾脏、外周血、骨髓	脾切除术、R2	PR	29

注 ^a^既往报道[8]；R：利妥昔单抗；CVP：环磷酰胺+长春新碱+甲泼尼龙；IR：伊布替尼+利妥昔单抗；ICE：异环磷酰胺+卡铂+依托泊苷；CHOP：环磷酰胺+阿霉素+长春新碱+甲泼尼龙；Len：来那度胺；Clb：苯丁酸氮芥；FC：氟达拉滨+环磷酰胺；R2：利妥昔单抗+来那度胺；CR：完全缓解；SD：疾病稳定；PD：疾病进展；PR：部分缓解

7例患者确诊时外周血淋巴细胞计数升高，中位值34.8（1.9～449.2）×10^9^/L，3例出现HGB下降，6例出现血小板降低。所有患者血清总蛋白、白蛋白水平均正常，均未检测到单克隆免疫球蛋白，均未发生溶血反应。本组病例中有2例患者评估疗效未达PR（例3和例4），治疗前外周血淋巴细胞计数、血清乳酸脱氢酶（LDH）和β_2_-微球蛋白（β_2_-MG）水平均明显升高，而HGB水平明显降低（表2）。

**表2:** 脾弥漫性红髓小B细胞淋巴瘤（SDRPL）患者的实验室检查结果

例号	淋巴细胞计数（×10^9^/L）	HGB（g/L）	PLT（×10^9^/L）	LDH（U/L）	β_2_-微球蛋白（ng/ml）
1	40.57	122	119	272	3 090
2	28.70	97	48	158	4 621
3	449.20	47	42	586	12 192
4^a^	77.85	81	83	1 455	6 096
5	20.50	152	65	155	1 558
6	1.80	132	98	136	2 170
7	29.00	139	141	348	4 150
8	40.80	136	140	321	2 425

注 ^a^既往报道[8]

2. 治疗及疗效：本组病例中位随访35.5（4～60）个月，仅1例在随访16个月时因疾病进展死亡。所有患者均行脾切除术，例1、2、6、7行脾切除术序贯免疫化疗后均达CR。例3确诊后予CVP（环磷酰胺+长春新碱+甲泼尼龙）、IR（伊布替尼+利妥昔单抗）、R-ICE（利妥昔单抗+异环磷酰胺+卡铂+依托泊苷）等方案化疗效果不佳，脾切除术后予R-CHOP（利妥昔单抗+环磷酰胺+阿霉素+长春新碱+甲泼尼龙）、来那度胺（Len）联合苯丁酸氮芥（Clb）化疗后获得长期生存。例5行脾切除术后B症状消失，但外周血淋巴细胞计数仍偏高，患者拒绝行免疫化疗，定期随访中。例8行脾切除术后外周血淋巴细胞仍高，予R2（利妥昔单抗+来那度胺）方案治疗后达PR。本研究组既往报道1例患者（例4）行脾切除术后1个月内进展为B细胞幼淋巴细胞白血病（B-PLL），先后予R-CHOP、FC（氟达拉滨+环磷酰胺）方案化疗效果不佳，后予Clb姑息治疗，16个月时死于疾病进展[8]。

3. 肿瘤细胞形态、脾脏病理、免疫表型及基因突变：所有患者外周血及骨髓中均可见肿瘤细胞，该类细胞胞体较大，核类圆形，胞质量丰富，易见宽基底的短毛刺状突起，呈极性分布（图1A、B）。脾脏病理示脾脏体积显著增大，可见形态均一的小淋巴细胞侵及红髓，弥漫浸润脾窦及脾索，白髓萎缩、消失，3例患者可见血湖形成（例2、4、6）。

例4初诊时骨髓中可见形态较均一的小淋巴细胞（图1C），在脾切除术后1个月出现外周血淋巴细胞显著升高，骨髓细胞形态学可见淋巴细胞胞体增大，核大而圆，部分细胞内可见明显的核仁（图1D）。该患者的免疫表型也发生了变化，CD23、CD25、CD200由强阳性转为阴性，CD38、CD180由弱阳性转为阴性，CD11c由阴性转为中等阳性，κ轻链由阴性转为阳性。

**图1:**
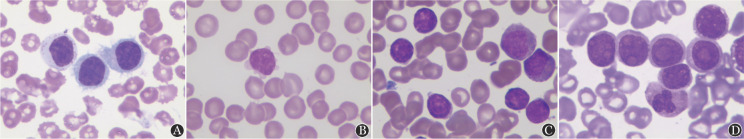
部分患者的肿瘤细胞形态（瑞氏染色，×1 000） A：例1外周血肿瘤细胞形态；B：例2外周血肿瘤细胞形态；C：例4脾切除前骨髓细胞形态；D：例4脾切除术后骨髓细胞形态

免疫组化：肿瘤细胞CD19、CD20、CD79α均阳性表达，6例Bcl-2阳性，2例CD11c阳性，3例IgD阳性37.5％，2例IgG阳性，1例IgM阳性；1例CD5阳性，所有患者CD10、CD25、CD123、CD103、Annexin-A1均阴性；中位Ki-67阳性率为20％（5％～60％）。例4的脾脏病理及免疫组化结果见图2。

**图2:**
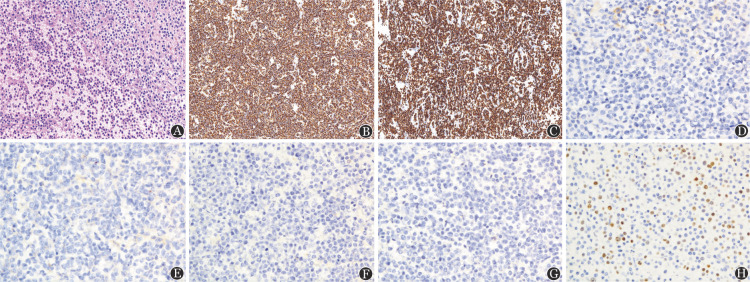
例4的脾脏病理HE染色（A）及免疫组化染色（B～H）结果 A：脾索与脾窦异型淋巴细胞弥漫分布，未见白髓，脾窦内弥漫分布异型淋巴细胞（×400）；B：CD20阳性（×200）；C：CD79a阳性（×200）；D：CD11c阴性（×400）；E：CD123阴性（×400）；F：CD25阴性（×400）；G：Annexin A1阴性（×400）；H：Ki-67阳性比例约60%（×400）

对8例患者脾脏组织行DNA测序，结果显示所有患者均未见BRAF V600E突变，1例有TP53缺失，1例有CCND3 P284A和MYC S146L突变，1例有CREBBP、TET2、BIRC3突变。

## 讨论

SDRPL是一种罕见的惰性NHL。目前对于该病的认识多来源于国外资料，国内报道很少。该病常累及脾脏、外周血和骨髓，可表现为淋巴瘤白血病期的特征，常有明显脾肿大，可伴B症状，消化道受累罕见[9]。

本研究患者男7例，女1例，临床分期均为Ⅳ期，累及脾脏、外周血和骨髓，未见外周淋巴结受累。部分病例可见脾门淋巴结受累，严重者可累及邻近肝及胰腺组织，使此疾病显示出一定的侵袭性。在中国知网资料总库、万方数据学术论文总库检索到SDRPL相关报道6篇[5]–[6],[10]–[13]，共10例患者，结合本组8例患者共18例，男女比例2∶1，确诊时中位年龄为58（40～73）岁，较国外报道年轻（中位年龄65.5岁）[14]。本研究显示，治疗后未达PR的患者治疗前外周血淋巴细胞计数、血清LDH和β_2_-MG水平更高，HGB更低，这些指标与肿瘤负荷相关，可能是预后的不良因素。

脾脏组织病理是诊断的金标准。脾脏常呈弥漫性、无结节性肿大，镜下可见单一形态的小淋巴细胞浸润红髓，白髓结构萎缩、消失；血窦破裂导致红细胞聚集，被肿瘤细胞包围，有时可形成典型的血湖[15]。SMZL的肿瘤细胞特点是明显的小结节型，围绕或取代白髓滤泡生发中心生长，使套区不清。HCL和HCL-v的浸润模式与SDRPL相似，单纯依靠脾脏病理很难区分，常需结合其他特征帮助识别。HCL常表现为显著的全血细胞减少，单核细胞减少，肿瘤细胞特异性表现为圆形或卵圆形核，胞质丰富，绒毛样突起沿细胞膜边缘分布，骨髓活检呈明显的网状纤维增生，并常导致骨髓干抽[16]。而HCL-v常表现为淋巴细胞增多，单核细胞易见，骨髓活检无明显的网状纤维化[17]。

本组病例的脾脏免疫表型为CD19、CD20、CD79α等B细胞抗原均阳性，CD10、CD25、CD123、CD103、Annexin-A1等均阴性；6例患者Bcl-2阳性，2例患者CD11c阳性，CD5多为阴性，与文献报道类似[18]。本组病例Ki-67多偏低，中位Ki-67阳性率为20％，但有1例短时间进展为B-PLL的患者Ki-67高达60％，提示Ki-67高表达可预示肿瘤的侵袭性强[8]。而HCL的免疫表型具有特异性，除表达B细胞抗原外，还表达CD11c、CD25、CD103、CD123、抗酒石酸酸性磷酸酶（TRAP）、Annexin A1[16]。HCL-v的免疫表型可与HCL部分相同，即CD11c和CD103阳性，但CD25、CD123和Annexin A1等阴性[17]。

HCL、HCL-v和SMZL中均有重现性基因突变的报道。几乎所有的HCL都携带BRAF V600E突变[16]。HCL-v约50％出现MAP2K1突变[17]。NOTCH2等突变在SMZL中被发现[19]。但目前对SDRPL分子机制的了解还非常有限。本组病例中1例患者发现了TP53缺失，1例出现CCND3和MYC基因突变，文献报道伴TP53或CCND3突变的患者可能具有更高的侵袭性[15],[20]。这表明SDRPL也可能存在特征性的突变模式和独特的致癌途径。

根据本组病例的临床及病理资料，结合相关文献报道，本文将常见的4种原发脾B细胞淋巴瘤的临床病理和分子学特征总结如下（表3）。

**表3:** 4种原发脾B细胞淋巴瘤的临床病理和分子学特征

特征	SDRPL	SMZL	HCL	HCL-v
血常规	常见白细胞增多，淋巴细胞增多，单核细胞易见	常见白细胞增多，淋巴细胞增多	常见全血细胞减少，严重的单核细胞减少，淋巴细胞增多罕见	常见白细胞增多，淋巴细胞增多，单核细胞正常
肿瘤细胞形态	染色质浓缩，核仁不可见，胞质嗜碱性，宽基底的绒毛状突起，极性分布	染色质浓缩，宽基底的多极性短突起，形态多样，可见浆细胞样分化	染色质呈海绵状，核圆形或凹形，核仁不明显或缺失；细的毛发状突起，呈环形分布，呈“煎鸡蛋”样	染色质浓缩，核仁明显，绒毛疏松，呈细突起，形态可多样
骨髓病理	弥漫性浸润髓窦与髓索	结节性浸润髓窦	间质浸润；可见网状纤维增加导致骨髓纤维化，常骨髓穿刺干抽	间质浸润，也常累及髓窦，无明显的网状纤维化
脾脏病理	弥漫侵犯红髓，伴有脾索和脾窦浸润，白髓萎缩	起源于脾边缘区，主要累及白髓	侵犯红髓，白髓萎缩	侵犯红髓，白髓萎缩
免疫表型	CD5（−），CD10（−），CD25（−），CD103（±），CD123（−），bcl-2（±），CyclinD1（−），Annexin A1（−），TRAP（−）	CD5（−），CD10（−），CD25（±），CD103（−），CD123（−），bcl-2（+），CyclinD1（−），Annexin A1（−），TRAP（−）	CD5（−），CD10（−），CD25（+），CD11c（+），CD103（+），CD123（+），cyclin D1（+），Annexin A1（+），TRAP（+）	CD5（−），CD10（−），CD25（−），CD11c（++），CD103（+），CD123（−），CyclinD1（−），Annexin A1（−），TRAP（−）
细胞遗传学	del(7q)，+3，+18	+3，del(7q)，del(17p)	+5，del(5q)，del(7q)，del(13q)	+5，del(17p)，del(17p)
突变基因	CCND3（突变频率约24%）[20]	NOTCH2（突变频率约20%）[19]	BRAF V600E（突变频率>80%）[16]	MAP2K1（突变频率约50%）[17]

注 SDRPL：脾弥漫性红髓小B细胞淋巴瘤；SMZL：脾边缘区淋巴瘤；HCL：毛细胞白血病；HCL-v：毛细胞白血病变异型

SDRPL呈惰性病程，多可长期生存，但目前尚无法治愈，最佳治疗方案还不明确。对于无明显临床症状或者年龄大的患者可以随访等待。对于有明显B症状、造血功能或重要脏器严重受累的患者，应尽早启动治疗，包括脾切除或免疫化疗[4]。本组病例中位随访35.5个月，仅1例在随访16个月时因疾病进展死亡。本组病例中例5仅接受了脾切除术，术后临床症状消失，随访18个月病情仍稳定。虽然脾切除术可以使病情得到一定程度的控制，但肿瘤细胞仍残留于骨髓及外周血，患者存在疾病进展的风险，脾切除术序贯免疫化疗可更好地控制患者病情，且总体耐受性良好。例1、2、6、7在脾切除术后接受利妥昔单抗联合CHOP样方案化疗后均达CR，疗效理想。例3、8在脾切除术后予以免疫化疗，虽然未达CR但均获得长期生存。

惰性淋巴瘤可向侵袭性更强的NHL转化，发生组织学转化的高危因素主要为HBV、EB病毒感染及MYC突变等[21]–[22]。当发生组织学转化时，应考虑强化化疗[8]。例4在脾切除术后1个月进展为B-PLL，予R-CHOP、FC等方案化疗后效果均不佳，该患者合并HBV感染，且病毒载量高，血清LDH、β_2_-MG显著升高，Ki-67升高，均提示预后不良。有研究报道，Bcl-2抑制剂联合布鲁顿酪氨酸激酶抑制剂伊布替尼治疗B-PLL可获得长期微小残留病阴性[23]。目前Bcl-2抑制剂已在CLL、急性髓系白血病等血液系统肿瘤的治疗中发挥重要作用，SDRPL中Bcl-2表达阳性也为运用Bcl-2抑制剂治疗提供了理论依据。

综上，SDRPL多见于>40岁男性，常表现为显著的脾肿大，外周血淋巴细胞计数增高。诊断的金标准是脾脏组织病理检查。外周血和骨髓中肿瘤细胞典型的细胞形态学、免疫表型也具有一定的诊断意义。SDRPL的治疗尚无共识，对无症状患者可以随访等待，有症状患者可通过脾切除术和（或）免疫化疗获得长期生存，但是发生组织学转化的患者可能具有更差的预后，需尽早给予更积极的干预措施。
